# Clinical and Radiological Characteristics to Differentiate Between EGFR Exon 21 and Exon 19 Mutations in Patients With Lung Adenocarcinoma: A Systematic Literature Review and Meta-Analysis

**DOI:** 10.7759/cureus.25446

**Published:** 2022-05-29

**Authors:** Andrés Felipe Herrera Ortiz, Mateo E Garland, Bassel Almarie

**Affiliations:** 1 Radiology, Fundación Santa Fe de Bogotá, Bogotá, COL; 2 Surgery, Beth Israel Deaconess Medical Center, Harvard Medical School, Boston, USA; 3 PPCR, Harvard T.H. Chan School of Public Health, Boston, USA

**Keywords:** lung adenocarcinoma, egfr mutation, exon 21, exon 19, computed tomography

## Abstract

Epidermal Growth Factor Receptor (EGFR) mutations in lung adenocarcinoma have been previously associated with specific clinical characteristics and Computed Tomography (CT) patterns. However, associations among individual EGFR mutations have not been evaluated. We aim to differentiate if the most common EGFR mutations (exon 21 and 19) are related to specific clinical characteristics or CT patterns. A systematic review and meta-analysis of 5 databases were conducted with literature from January 2002 to July 2021. Eligible studies were of an experimental or observational design that included lung adenocarcinoma patients with confirmed EGFR exon mutations (21 and 19) and associated clinical characteristics and CT imaging patterns. Quality was assessed using the QUADAS-2 tool. The association between clinical and CT patterns and EGFR exon mutations 21 and 19 was evaluated using odds ratios (OR) and then pooled and analyzed with a fixed or random-effects model. This study follows the preferred reporting items for systematic review and meta-analysis (PRISMA) guidelines.

A total of 12 retrospective diagnostic accuracy studies were included. Pooled analysis showed that characteristics such as absence of smoking status (OR 1.29 [95% CI 0.97 - 1.70]), and female sex (OR 1.23 [95% CI 0.83 - 1.82]); and CT patterns such as Ground Glass Opacities (GGO) (OR 1.03 [95% CI 0.78 -1.34]), air bronchogram (OR 0.78 [95% CI 0.44 -1.39]), pleural retraction (OR 0.83 [95% CI 0.53 - 1.28]), and spiculation (OR 0.80 [95% CI 0.48 - 1.31]) were not significantly associated to a specific mutation. Specific EGFR exon 21 and 19 mutations cannot be differentiated through characteristics (absence of smoking status and female sex) or radiological patterns (GGO, air bronchogram, pleural retraction, and speculation). There is limited data to assess if early disease stage or vascular convergence aids in differentiating exon 21 from 19 mutations in patients with lung adenocarcinoma.

## Introduction and background

Lung cancer is one of the most commonly diagnosed cancers globally and the leading cause of cancer-related mortality. It is divided into small cell lung cancer and non-small cell lung cancer (NSCLC). NSCLC accounts for approximately 85% of all lung cancers and is subtyped into adenocarcinoma, squamous cell carcinoma, and large cell carcinoma. Adenocarcinoma is the most common form of lung cancer and the most common subtype of NSCLC, accounting for 40% of all NSCLC occurrences [1]. The two most common epidermal growth factor receptor (EGFR) mutations, and L858R point mutation in exon 21 and exon 19 deletions, occur in roughly 90% of all mutation-positive NSCLC tumors [2]. These two mutations are most common in lung adenocarcinomas, with an L858R point mutation in exon 21 in 40% and Exon 19 deletion in 45% of all lung adenocarcinomas. Both mutations are referred to as sensitizing EGFR mutations [3-5]. 

Detecting these two oncogenic driver mutations has become essential in the treatment of NSCLC, specifically adenocarcinoma [6], as both mutations are sensitive to drugs that target EGFR [2], and screening for these mutations predict which patients will respond to therapy [7]. Advances in research demonstrated that EGFR mutations are linked to specific risk factors such as the absence of smoking and female sex, and radiological imaging features such as ground-glass opacities (GGO), air bronchogram, vascular convergence, pleural retraction, spiculation [5,8,9].

The existing literature revealed that these two mutations share clinical and radiological features in patients with adenocarcinoma. For instance, it has been described that L858R point mutation in exon 21 had a higher association with the absence of smoking status and female sex. In contrast, exon 19 deletions are usually presented more in women, tumors with smaller maximum diameter, a higher proportion of GGO, and pleural retraction [10-12]. However, the exact clinical and radiological features and their association with a specific mutation remain disputable. Thus, we performed a systematic review and meta-analysis to investigate if specific clinical and computed tomography (CT) patterns could help to differentiate between EGFR in exon 21 and exon 19 in patients with lung adenocarcinoma.

## Review

Material and methods

This systematic literature review and meta-analysis was conducted and reported following the Preferred Reporting Items for Systematic Review and Meta-analysis (PRISMA) [13]. 

Eligibility Criteria

We included observational studies and clinical trials conducted on patients with lung adenocarcinoma with EGFR mutation in exon 21 and exon 19. The diagnosis was based on a CT scan to identify the imaging patterns of the growth and biopsy or cytology to confirm results and detect EGFR mutation in exon 21 and exon 19. There were no restrictions on disease stage, age, sex, geographical region, or hospitalization status. We excluded articles that lacked clinical and CT characteristics variables needed to calculate the odds ratio (OR). These articles included patients who received radiotherapy, chemotherapy, biological therapy, or surgery prior to CT scan and articles with a high risk of bias based on the Quality Assessment of Diagnostic Accuracy Studies-2 (QUADAS-2).

Sources of Information

We systematically searched for studies on humans published between January 2002 and July 2021 to evaluate the association between clinical and radiological characteristics of lung adenocarcinomas and EGFR mutation of exon 21 and exon 19. We limited the query to studies published in English, Spanish, and French. The literature search was performed in MEDLINE, EMBASE, VHL, the African Index Medicus, and google scholar. Additional methods were applied to identify further articles, including the snowball technique and hand-searching of three selected high-impact journals (European Journal of Radiology, Radiology, American Journal of Roentgenology) [14,15].

Search

We used the following search strategy and keywords: “Epidermal growth factor receptor” OR “EGFR” OR “epidermal growth factor receptor mutation” AND “Carcinoma, Non-Small-Cell Lung” OR “NSCLC” OR “non-small cell lung cancer” OR “lung adenocarcinoma” OR “lung cancer” OR “lung carcinoma” AND “Tomography, X-Ray Computed” OR “Tomography, Spiral Computed” OR computed tomography” OR CT” AND “Biopsy” OR “cytology”.

Study Selection

The articles identified by the search strategy were uploaded to Mendeley, a citation database program for review and selection. Duplicates were removed, and the remaining articles were screened by abstracts and titles against inclusion and exclusion criteria using pre-defined questions. Potentially relevant articles were evaluated in their entirety and subjected to quality assessment. The entire process was carried out by two authors independently. Disagreements were discussed and resolved by consensus. In the case of two articles or more with the same population but different research questions, the article with the largest sample size was considered.

Data Extraction and Missing Data

The data from all the articles were extracted by two authors independently. Information extracted was registered in a qualitative data extraction table that included the author’s name, year of publication, country, study type, number of patients, mean age of the participants, specific exon mutation, disease stage, index test, reference standard used, and evaluated clinical and CT pattern. 

An additional quantitative data extraction table was formed to register the presence or absence of each of the clinical and CT variables in EGFR mutation in exon 21 and EGFR mutation exon 19. The quantitative data extraction table registered the following data: true positive (TP), false positive (FP), false negative (FN), and true negative (TN), as demonstrated in (Table 1). In case of missing or inconsistent data, corresponding authors were contacted. The information was missing and not included if the authors did not respond. Two independent authors reviewed qualitative and quantitative data extraction tables to ensure a high quality of data extraction. Disagreements were solved by consensus. 

**Table 1:** Example of Quantitative Data Extraction Table

	Exon 21	Exon 19
Positive radiological/clinical characteristic	TP	FP
Negative radiological/clinical characteristic	FN	TN

Outcomes

The primary outcome was to evaluate the association of each of the specific clinical and CT patterns (absence of smoking status, female sex, early disease stage, GGO, air bronchogram, pleural retraction, spiculation, and vascular convergence) to EGFR mutation in exon 21 as compared to EGFR mutation exon 19 among patients with lung adenocarcinoma.

Quality Assessment

To ascertain the validity and quality of included studies, each study was assessed using the QUADAS-2 tool [16]. Only studies with a low risk of bias and low concern regarding applicability were considered for inclusion. A traffic light plot was built using the Robvis tool [15].

Effect Measures

Each clinical and CT pattern was crossed with exon 21 and exon 19 in a two-by-two table to obtain the variables TP, FP, FN, and TN. Based on these variables, the OR of each article was calculated (Table 1), then pooled using forest plots to obtain an overall effect.

Statistical Analysis 

All statistical analyses were performed using STATA 17 (StataCorp LLC, College Station, TX). Statistical heterogeneity of the studies was evaluated using Cochran’s Q statistic and quantified by the I2 value [15]. Cochran’s Q test of less than 0.05 indicated a high degree of heterogeneity. A random-effect model was established for I2 values higher than 50%, and a fixed-effect model for I2 lower than 50%. A random-effect model was chosen in case of discrepancies between Cochran’s Q test and the I2 test.

Publication bias was assessed using Egger’s test [15], with a P value of less than 0.05 as indicative of publication bias. Forest plots were based on OR value of each of the clinical and CT patterns. All results were evaluated for robustness by performing a sensitivity analysis.

Results

Search Results

**Figure 1:**
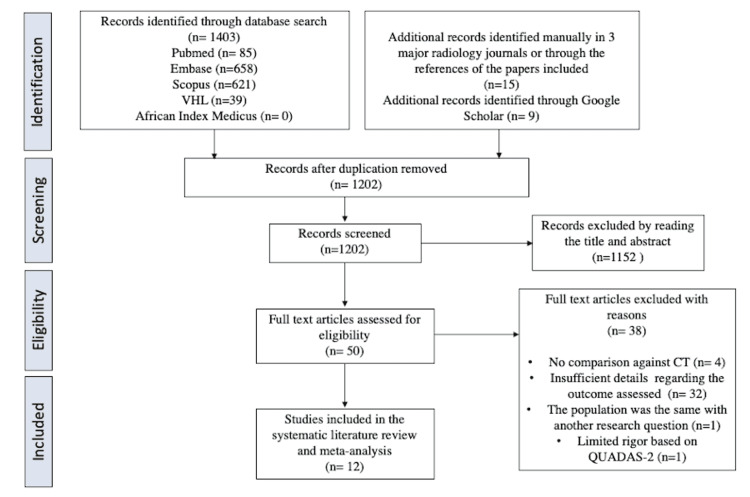
PRISMA Flow Diagram CT: Computed tomography

The literature search yielded 1202 articles. After reviewing the titles and abstracts, we excluded 1152 articles for not meeting the inclusion criteria. The remaining 50 articles were reviewed and read in their entirety. Based on this review, we excluded four articles for lacking comparison of EGFR mutation to CT findings, 32 articles for lacking the variables needed to calculate the OR, 1 article for having the same population but different research question from an article already included, and 1 article for high risk of bias based on QUADAS-2 tool. The remaining 12 articles were included in the meta-analysis. The PRISMA flow diagram is illustrated in Figure 1.

Summary of Studies

The final analysis included 12 retrospective diagnostic accuracy studies [11,17-27]. These studies assessed a total of 1953 patients, with sample sizes ranging from 71 to 351 per study. The mean age of the patients ranged between 56 and 66 years. Mutations were present: Exon 21 missense in 850 patients, exon 21 L858R mutation in 152 patients, exon 19 deletion in 863 patients, and exon 19 missense in 88 patients. The qualitative synthesis of the articles included is demonstrated in Table 2.

**Table 2:** Qualitative Data Extraction

Author	Year	Country	Study Type		Number of Patients	Mean Age	Exon Mutation	Disease Stage	Index Test (Slice Thickness)	Reference Standard Test (Method Used)	Clinical and CT Pattern Described
Lee et al. [20]	2013	Korea		Retrospective diagnostic accuracy study	78	63	Exon 21 missense, Exon 19 deletion	Stage I-III	CT (1-5mm)	Biopsy (PCR)	Absence of smoking, female sex
Hsu et al. [22]	2014	Taiwan		Retrospective diagnostic accuracy study	71	63	Exon 21 missense, Exon 19 deletion	Stage III-IV	CT (1-5mm)	Biopsy (PCR)	Absence of smoking, female sex, GGO, air bronchogram, spiculation
Qin et al. [23]	2018	China		Retrospective diagnostic accuracy study	351	64	Exon 21 missense, Exon 19 deletion	Not described	CT (1.25-5mm)	Biopsy (PCR)	Absence of smoking, female sex, air bronchogram
Hasegawa et al. [24]	2016	Japan		Retrospective diagnostic accuracy study	100	66	Exon 21 missense, Exon 19 deletion	Stage I-IV	CT (1 mm)	Biopsy (PCR)	GGO
Dai et al. [25]	2015	China		Retrospective diagnostic accuracy study	104	58,3	Exon 21 missense, Exon 19 deletion	Stage I	CT (2 mm)	Biopsy (PCR)	Absence of smoking, female sex
Yang et al. [17]	2019	China		Retrospective diagnostic accuracy study	290	56.75 years	Exon 21 missense, Exon 19 deletion, Exon 19 missense	Not described	CT (2 mm)	Biopsy (PCR)	GGO
Suh et al. [21]	2018	Korea		Retrospective diagnostic accuracy study	281	Not described	Exon 21 missense, Exon 19 deletion	Not described	CT (1-2.5 mm)	Biopsy (PCR)	GGO
Hong et al. [26]	2015	Korea		Retrospective diagnostic accuracy study	111	63	Exon 21 missense, Exon 19 deletion	Stage I-IV	CT (1-3mm)	Biopsy (PCR)	Absence of smoking, female sex, GGO
Zou et al. [18]	2017	China		Retrospective diagnostic accuracy study	95	60,11	Exon 19 deletion, L858R mutation in exon 21	Stage I-II	CT (1mm)	Biopsy (PCR)	GGO, air bronchogram, pleural retraction, spiculation, vascular convergence
Park et al. [19]	2016	Japan		Retrospective diagnostic accuracy study	111	62,1	Exon 19 deletion, L858R mutation in exon 21	Stage III-IV	CT (Not described)	Biopsy (PCR)	GGO, air bronchogram
Shi et al. [28]	2018	China		Retrospective diagnostic accuracy study	272	59,39	Exon 21 missense, Exon 19 deletion	Not described	CT (Not described)	Biopsy (PCR)	Absence of smoking, female sex, GGO, air bronchogram, pleural retraction, spiculation
Hsu et al. [27]	2011	Taiwan		Retrospective diagnostic accuracy study	89	59	Exon 19 deletion, L858R mutation in exon 21	Stage I	CT (Not described)	Biopsy (PCR)	Absence of smoking, female sex, GGO

Risk of Bias Within Studies

**Figure 2:**
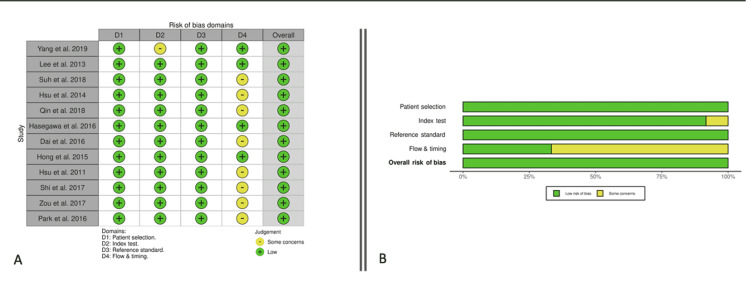
Quality Assessment of Included Articles

The analysis showed an overall low or acceptable risk of bias in all included studies. Using the QUADAS-2 tool to assess quality, nine studies were rated as having some concern of bias in only 1 of the 4 core domains, and three were considered to have a low risk of bias in all four core domains [16]. Figure 2 demonstrates the overall risk of bias assessment.

Clinical Characteristics to Differentiate Between EGFR Mutation in Exon 21 and Exon 19

**Figure 3:**
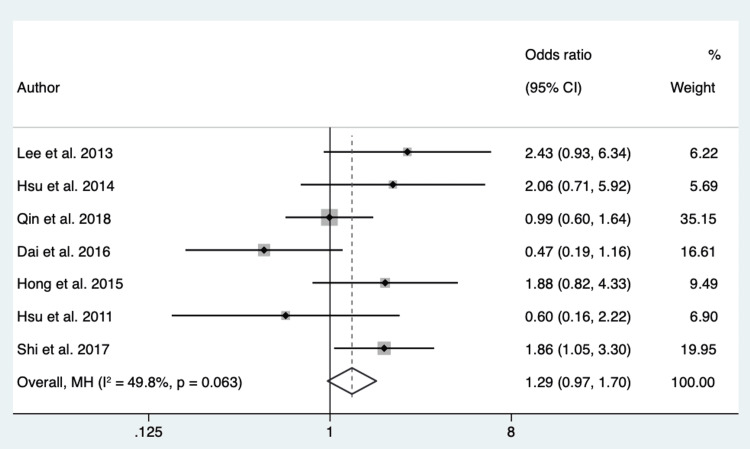
Forest Plot of Absence of Smoking Status in Respect to EGFR in Exon 21 vs. Exon 19 Source: References [20,22,23,25-27].

Absence of Smoking: One thousand seventy-six patients from 7 different studies were pooled to assess the relationship between the absence of smoking status and EGFR mutation in exon 21 and EGFR mutation in exon 19. The P-value of Cochran’s Q test was 0.06, and the I2 value was 49.8%. We considered low heterogeneity in the data and used a fixed-effect model. The overall effect showed an OR of 1.29 (95% CI 0.97 - 1.70) (Figure 3).

**Figure 4:**
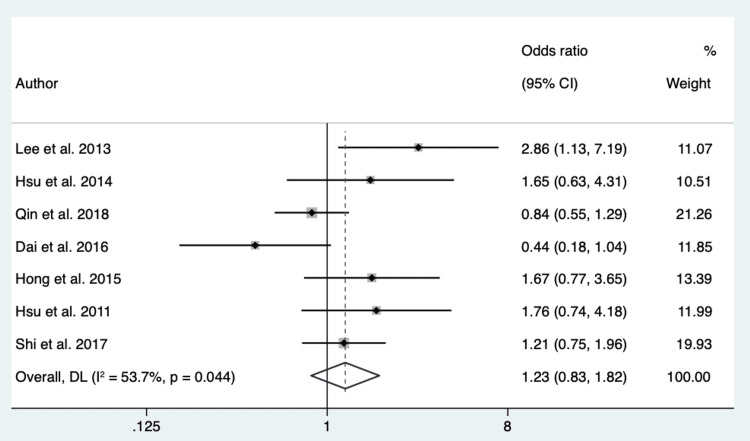
Forest Plot of Female Sex in Respect to EGFR Mutation in Exon 21 vs. Exon 19 Source: References [20,22,23,25-27].

Female Sex: One thousand seventy-six patients from 7 different studies were pooled to evaluate the association of female sex to EGFR mutation in exon 21 compared to EGFR mutation in exon 19. The P-value of Cochran’s Q test was 0.044, and the I2 value was 53.7%. We considered high heterogeneity in the data and used a random-effect model based on these results. The overall effect showed an OR of 1.23 (95% CI 0.83 - 1.82) (Figure 4).

Early Disease Stage: No articles evaluated the presence of early disease stage and EGFR mutation in exon 21 or exon 19. Therefore, no statistical analysis was performed.

Radiological Imaging Patterns to Differentiate Between EGFR Mutation in Exon 21 and Exon 19

**Figure 5:**
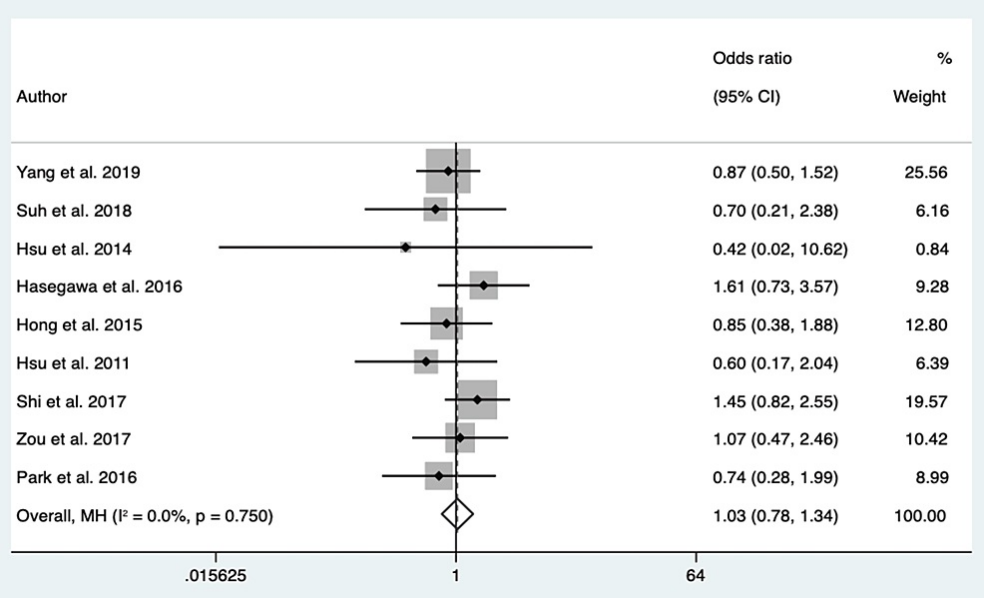
Forest Plot of GGO in Respect to EGFR Mutation in Exon 21 vs. Exon 19 Source: References [17,21,22,24,26-28,18,19].

GGO: One thousand four hundred twenty patients from 9 different studies were pooled to assess the role of the imaging pattern, GGO, to differentiate EGFR mutation in exon 21 from EGFR mutation in exon 19. The P-value of Cochran’s Q test was 0.75, and the I2 value was 0%. We considered the data homogeneous and used a fixed-effect model based on these results. The overall effect showed an OR of 1.03 (95% CI 0.78 -1.34) (Figure 5).

**Figure 6:**
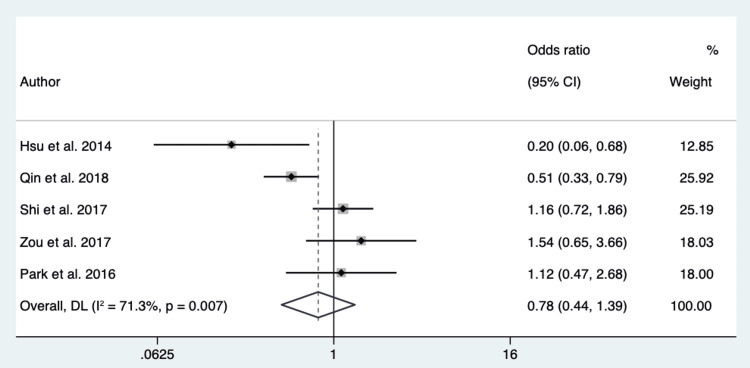
Forest Plot of Air Bronchogram to Differentiate Between EGFR Mutation in Exon 21 and Exon 19 Source: References [22,23,28,18,19].

Air Bronchogram: Nine hundred patients from 5 different studies were pooled to assess if an air bronchogram differentiates EGFR mutation in exon 21 from EGFR mutation exon 19. The P-value of Cochran’s Q test was 0.007, and the I2 value was 71.3%. We considered high heterogeneity in the data based on these results, and a random-effect model was used. The overall effect showed an OR of 0.78 (95% CI 0.44 -1.39) (Figure 6).

**Figure 7:**
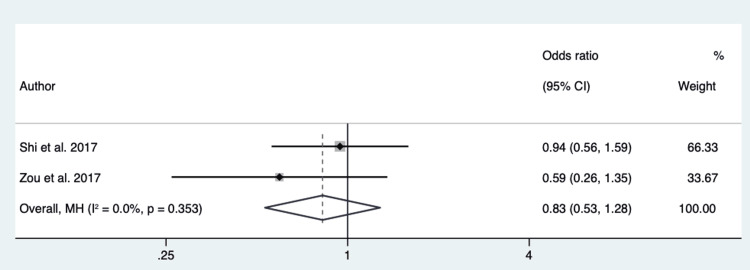
Forest Plot of Pleural Retraction in Respect to EGFR in Exon 21 vs. Exon 19 Source: References [28,18].

Pleural retraction: Three hundred sixty-seven patients from 2 different studies were pooled to assess the role of pleural retraction in differentiating EGFR mutation in exon 21 from EGFR mutation in exon 19. The P-value of Cochran’s Q test was 0.35, and the I2 value was 0%. We considered low heterogeneity in the data and used a fixed-effect model based on these results. The overall effect showed an OR of 0.83 (95% CI 0.53 - 1.28) (Figure 7).

**Figure 8:**
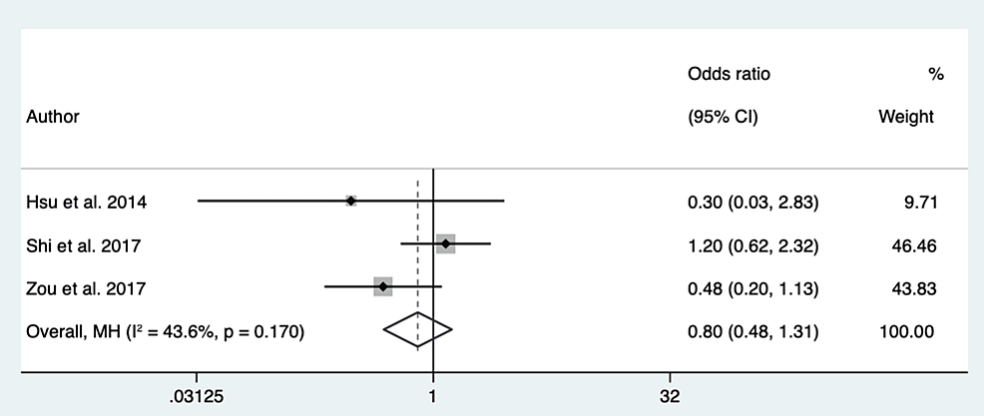
Forest Plot for Spiculation in Respect to EGFR in Exon 21 vs. Exon 19 Source: References [22,28,18].

Spiculation: Four hundred thirty-eight patients from 3 different studies were pooled to assess if the variable spiculated margins have a role in differentiating EGFR mutation in exon 21 from EGFR mutation in exon 19. The P-value of Cochran’s Q test was 0.17, and the I2 value was 43.6%. The results suggested low heterogeneity in the data. Therefore, a fixed-effect model was used. The overall effect showed an OR of 0.80 (95% CI 0.48 - 1.31) (Figure 8).

Vascular Convergence: We only found one study of 95 patients evaluating the variable vascular convergence. Thus, no forest plot was performed. The OR was 0.61 (95% CI 0.20 - 1.86).

Sensitivity Analysis

Sensitivity analysis was performed for all the forest plots showing no change in the overall effect upon removal articles. The only two exceptions were the forest plots for spiculation and the absence of smoking status. After removing the article published by Shi et al., the effect size of spiculation changes to become protective. For the absence of smoking status, the effect became a risk factor for EGFR mutation in exon 21 when some studies were removed.

Publication Bias

Using Egger’s test, no publication bias was detected in all forest plots. The results of Egger’s test are demonstrated in Table 3. 

**Table 3:** Egger's Test Results For Publication Bias

Outcome Assessed	P value
Absence of smoking status to differentiate between EGFR in exon 21 and exon 19	0.88
Female Sex to differentiate between EGFR in exon 21 and exon 19	0.35
GGO to differentiate between EGFR in exon 21 and exon 19	0.23
Air bronchogram to differentiate between EGFR in exon 21 and exon 19	0.51
Pleural retraction to differentiate between EGFR in exon 21 and exon 19	0.35
Spiculation to differentiate between EGFR in exon 21 and exon 19	0.19

Discussion

While most present-day evidence associates EGFR mutation to specific clinical characteristics and CT patterns, a paucity of evidence is available to evaluate individual types of EGFR mutation for these characteristics and radiological patterns. This systematic literature review and meta-analysis revealed that clinical characteristics such as the absence of smoking, female sex, and early disease stage, and CT patterns such as GGO, air bronchogram, pleural retraction, spiculation, and vascular convergence have no significant value in differentiating EGFR mutation in exon 21 from EGFR mutation in exon 19. 

The present study’s findings settle conflicting results of previous studies, some of which linked the detection of EGFR mutation in exon 21 to the female sex and other factors such as the absence of smoking [10,12]. In contrast, others suggested that EGFR mutation in exon 19 was predominately detected among females and associated with other factors such as pleural retraction and a higher proportion of GGO [11,12]. 

In a study published by Shi et al., the authors found that emphysema, tumors with a diameter of less than 34.5mm, and fibrosis were associated with EGFR mutation in exon 19. The study’s findings compared exon 19 mutation and EGFR wild type. When extrapolated, such findings lack external validity to differentiate between EGFR mutation in exon 19 and other exons [28]. The clinical characteristics and CT patterns are associated with the two common EGFR mutations, exon 21 and 19. However, when one of these EGFR mutations, exon 21 or 19, is compared with EGFR wild type, the findings suggest that a mutation in exon 21 or 19 is associated with the clinical characteristics or CT patterns.

In a previous study, Qin et al. suggested that air bronchogram had a stronger association with EGFR mutation in exon 19 than EGFR mutation in exon 21. Nevertheless, multiple comparisons in the study have possibly led to false-positive results. Each calculation of the p-value with a set threshold of 0.05 has a 5% chance of a false-positive outcome. Repetitive calculations, aimed for multiple comparisons, can stack this value to a sum of 1.00, causing false-positive results [23]. 

GGO pattern has been previously associated with EGFR mutations by multiple studies. However, contradicting results can be seen in the literature when comparing individual exons. Lee et al. suggested that the GGO pattern frequently presents in tumors with exon 21 mutations than exon 19 [20]. However, in a study published three years later, Hong et al. showed no significant differences in GGO proportion in lung adenocarcinoma with EGFR 21 and 19 mutations [26]. Although sample sizes and methodology were similar, a specific variation in histologic subtypes can explain the difference. Our pooled results support the hypothesis that the GGO pattern is present in both 21 and 19 mutations without a significant difference, and it might be worthwhile to explore histologic patterns in addition to exon mutation.

Identifying EGFR mutation in lung cancer is crucial for appropriate treatment with EGFR tyrosine kinase inhibitors. Recent studies showed differences in outcomes after EGFR tyrosine kinase inhibitors treatment between exon 21 and 19 mutations [29,30]. Although the literature supports that clinical and CT patterns may help physicians suspect the presence of EGFR mutation, our study suggests that differentiation between EGFR 21 and 19 mutations through clinical and CT patterns alone is not advisable for clinicians.

Nonetheless, there are significant limitations to this study. While the meta-analysis had a thorough search strategy, including database search, hand search, and snowball methods, the studies included were limited by their retrospective design, prone to selection bias and confounders. The sample size was large, with many CT patterns and clinical variables. However, the variables “absence of smoking” and “spiculated margins” lacked robustness when performing sensitivity analysis. In light of more vital evidence, the value of these two variables may change.

Moreover, the variables “early disease stage” and “vascular convergence” lacked a sufficient sum to be statistically evaluated. Therefore, the association of these variables to EGFR mutation in exon 21 or exon 19 remains unclear and must be further researched. The statistical power of Egger’s test to detect publication bias was not considered high-powered as none of the forest plots generated included more than 10 articles. The absence of publication bias may also represent false-negative outcomes [15]. Lastly, the studies included in this analysis were primarily from Korean, Chinese, and Japanese literature. Therefore, our results should be carefully extrapolated to other populations.

## Conclusions

Specific EGFR exon 21 and 19 mutations cannot be unequivocally differentiated through characteristics such as the absence of smoking status and female sex or radiological patterns such as GGO, air bronchogram, pleural retraction, and spiculation. There is limited data to assess if early disease stage or vascular convergence aids in differentiating exon 21 from 19 mutations in patients with lung adenocarcinoma.
